# Family history of stroke and cardiovascular diseases in early-onset cryptogenic ischaemic stroke

**DOI:** 10.1093/esj/aakag013

**Published:** 2026-03-07

**Authors:** Maximilian C Sihvo, Pauli Ylikotila, Marialuisa Zedde, Rosario Pascarella, Tomi Sarkanen, Dalius Jatužis, Kristina Ryliškienė, Bettina von Sarnowski, Radim Licenik, Phillip Ferdinand, Janika Kõrv, Liisa Kõrv, Alessandro Pezzini, Ana Catarina Fonseca, André Paula, Patricia Martínez-Sánchez, Nilufer Yesilot, Annette Fromm, Ulrike Waje-Andreassen, Petra Redfors, Katarina Jood, Juha Huhtakangas, Tiina Sairanen, Marja Hedman, Pekka Jäkälä, Hugo ten Cate, Eva Gerdts, Mika Lehto, Juha Sinisalo, Steven J Kittner, Braxton D Mitchell, Arne G Lindgren, Andreea Ilinca, Jukka Putaala, Liisa Tomppo

**Affiliations:** Helsinki University Central Hospital and University of Helsinki, Helsinki, Finland; Department of Neurology, Turku University Hospital and University of Turku, Finland; Neurology Unit, Stroke Unit, Azienda Unità Sanitaria Locale, IRCCS Reggio Emilia, Italy; Neuroradiology Unit, Ospedale Santa Maria della Misericordia, Rovigo, Italy; Department of Neurology, Tampere University Hospital, Wellbeing Services County of Pirkanmaa and Faculty of Medicine and Health Technology, Tampere University, Tampere, Finland; Department of Neurology and Neurosurgery, Institute of Clinical Medicine, Faculty of Medicine, Vilnius University, Vilnius, Lithuania; Department of Neurology and Neurosurgery, Institute of Clinical Medicine, Faculty of Medicine, Vilnius University, Vilnius, Lithuania; Department of Neurology, University Medicine Greifswald, Greifswald, Germany; Acute Stroke Centre, North West Anglia NHS Foundation Trust, Peterborough City Hospital, Peterborough, United Kingdom; Neurosciences, University Hospitals of North Midlands NHS Trust, Stoke-on-Trent, United Kingdom; Department of Neurology and Neurosurgery, University of Tartu, Tartu, Estonia; Department of Neurology and Neurosurgery, University of Tartu, Tartu, Estonia; Department of Medicine and Surgery, University of Parma, Parma, Italy; Stroke Care Program, Parma University Hospital, Parma, Italy; Hospital Santa Maria, Faculty of Medicine, University of Lisbon, Lisbon, Portugal; Hospital Santa Maria, Faculty of Medicine, University of Lisbon, Lisbon, Portugal; Department of Neurology, Torrecardenas University Hospital, University of Almería, Almería, Spain; Department of Neurology, Istanbul University Faculty of Medicine, Istanbul, Turkey; Department of Neurology, Haukeland University Hospital, Bergen, Norway; Department of Neurology, Haukeland University Hospital, Bergen, Norway; Department of Neurology, Sahlgrenska University Hospital and Department of Clinical Neuroscience, Institute of Neuroscience and Physiology, Sahlgrenska Academy at University of Gothenburg, Gothenburg, Sweden; Department of Neurology, Sahlgrenska University Hospital and Department of Clinical Neuroscience, Institute of Neuroscience and Physiology, Sahlgrenska Academy at University of Gothenburg, Gothenburg, Sweden; Department of Neurology, Oulu University Hospital and University of Oulu, Oulu, Finland; Helsinki University Central Hospital and University of Helsinki, Helsinki, Finland; Heart Center, Kuopio University Hospital, Kuopio, Finland; Neurocenter Neurology, Kuopio University Hospital, Finland and University of Eastern Finland, Kuopio, Finland; Department of Internal Medicine, and Thrombosis Expert Center, Maastricht University Medical Center and CARIM school for cardiovascular diseases, Maastricht, The Netherlands; Center for Research on Cardiac Disease in Women, Department of Clinical Science, University of Bergen, Bergen, Norway; Department of Cardiology, Heart and Lung Center, Helsinki University Hospital and University of Helsinki, Helsinki, Finland; Department of Cardiology, Heart and Lung Center, Helsinki University Hospital and University of Helsinki, Helsinki, Finland; Department of Neurology, University of Maryland School of Medicine, Baltimore, MD, United States; Department of Medicine, University of Maryland School of Medicine, Baltimore, MD, United States; Department of Clinical Sciences Lund, Neurology, Lund University, Lund, Sweden; Department of Clinical Sciences Lund, Neurology, Lund University, Lund, Sweden; Department of Neurology, Skåne University Hospital, Malmö, Sweden; Helsinki University Central Hospital and University of Helsinki, Helsinki, Finland; Helsinki University Central Hospital and University of Helsinki, Helsinki, Finland

**Keywords:** ischaemic stroke, cryptogenic, young stroke, PFO, family history

## Abstract

**Background:**

Familial aggregation of stroke is well-documented, yet few studies have examined associations between stroke subtypes—particularly early-onset cryptogenic ischaemic stroke (eCIS)—and broader family history (FH) of cardiovascular disease. Such associations may provide insights into underlying etiologic mechanisms.

**Methods:**

In this multicentre case–control study, we included eCIS patients aged 18–49 years and matched stroke-free controls. We analysed the association between FH of stroke, venous thromboembolism (VTE), coronary artery disease (CAD), aneurysms and eCIS using multivariable logistic regression, with a subgroup analysis stratifying patients by high-risk patent foramen ovale (HR-PFO).

**Results:**

We enrolled 508 eCIS patients (182 [36%] with HR-PFO) and 520 controls. Compared with controls, patients more frequently reported FH of stroke among first-degree relatives (FDR) (20% vs. 14%, *P* = .01) and grandparents (47% vs. 39%, *P* = .01), FH of early-onset stroke among FDR (5% vs. 2%, *P* = .01) and FH of early-onset VTE among FDR (5% vs. 2%, *P* = .003). In adjusted analyses, eCIS was associated with FH of stroke among FDR (OR 1.50; 95% CI, 1.04–2.16) and grandparents (1.50; 1.12–1.99), with FH of early-onset stroke among FDR (2.36; 1.11–5.04); and with FH of early-onset VTE among FDR (3.45; 1.47–8.13). eCIS was also associated with FH of VTE among FDR (1.80, 1.09–2.98) in the presence of HR-PFO. FH of CAD or aneurysms was not associated with eCIS.

**Conclusion:**

FH of stroke and VTE, particularly early-onset events and in the presence of HR-PFO, are associated with eCIS. These findings support familial predisposition and highlight prothrombotic mechanisms in eCIS.

**Clinical trial registration:**

www.clinicaltrials.gov/study/NCT01934725

## Graphical Abstract

**Graphical Abstract:**
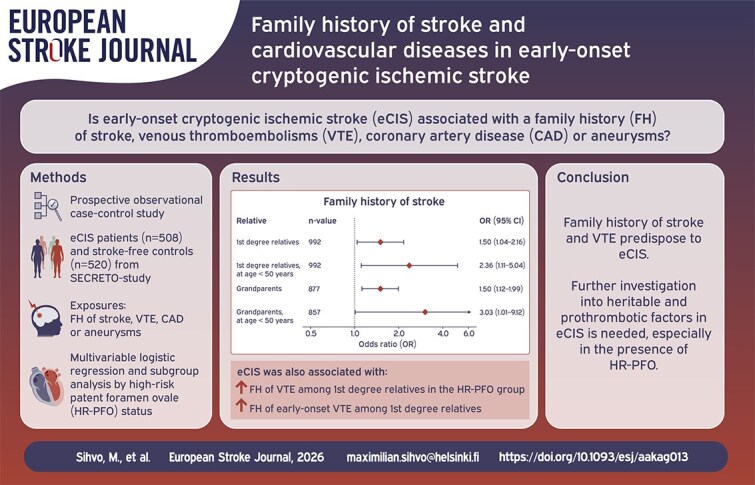

## Introduction

Ischaemic stroke in young adults is a rising health concern, given its increasing incidence, substantial morbidity and mortality.^1^ Early-onset cryptogenic ischaemic strokes (eCIS), defined as ischaemic stroke in adult patients younger than 50 years without a clearly identifiable cause despite a comprehensive diagnostic workup, account for up to 50% of ischaemic strokes in patients aged 18–49 years.^2–5^ Many eCIS patients are diagnosed with high-risk patent foramen ovale (HR-PFO) that could predispose to ischaemic stroke through paradoxical embolism.^6^

Several studies have reported associations between stroke, along with its subtypes, and a family history (FH) of stroke.^7–12^ However, few studies have comprehensively examined the relationship between stroke and a broader FH of cardiovascular disease (CVD) across stroke subtypes, particularly in eCIS. Establishing such associations may offer valuable insights into underlying etiologic mechanisms.

We hypothesize that CVD clusters within families of patients diagnosed with eCIS, especially early-onset disease. We further hypothesize that early-onset venous thromboembolism (VTE) in relatives may indicate inherited thrombophilia or shared prothrombotic determinants. In young adults with cryptogenic stroke, particularly those with HR-PFO, venous thrombosis may lead to a paradoxical embolism. Therefore, the objective of this international multicentre study was to evaluate the FH of stroke, VTE, coronary artery disease (CAD) and aortic or cerebrovascular arterial aneurysms in eCIS patients and controls, and to further stratify patients by HR-PFO status.

## Patients and methods

### Ethics statement

The study was approved by the Ethics Committee of the Helsinki and Uusimaa Hospital District (362/13/03/00/2012) and local ethics committees at each recruiting centre. Each participant provided written informed consent. The study follows the Strengthening the Reporting of Observational Studies in Epidemiology guidelines.^13^

### Study population

SECRETO (Searching for Explanations for Cryptogenic Stroke in the Young: Revealing the Etiology, Triggers, and Outcome; NCT01934725) is an international prospective multicentre case–control study of young adults presenting with a first-ever imaging-positive eCIS. The study included patients aged 18–49 after a thorough clinical assessment, along with age (±5 years), sex and regionally matched stroke-free controls from 19 study sites across Europe between 2013 and 2022. The study protocol has been described in more detail before.^14^

Each participant’s complete clinical history was obtained through a structured interview and medical record review including demographic details (age and sex) and the following well-established stroke risk factors: previous CVDs (CAD, congestive heart failure, myocardial infarction, atrial fibrillation or peripheral artery disease), hypertension, diabetes mellitus, hypercholesterolemia, current smoking, abdominal obesity, physical inactivity, heavy alcohol use, poor diet, stress, depression and education level.^15^^,^^16^ Detailed definitions for each variable are listed in Table S1.

Presence of an HR-PFO, defined as a patent foramen ovale with an atrial septal aneurysm or a large-sized right-to-left shunt (≥25 microbubbles crossing the atrial septum) among patients, was assessed through a transthoracic and a transesophageal echocardiogram.^14^

Information on participants’ FH was obtained through a structured questionnaire. We included the FH of any stroke, VTE, CAD and aortic or cerebrovascular aneurysms among first-degree relatives (FDR)—including offspring, siblings and parents—and grandparents. The age of family members at the onset of the disease was recorded, and we defined disease with onset before age 50 as early-onset. We created composite variables combining FH among FDR and grandparents and considered FH to be positive if any family member in that group was reported to have the disease, even if there was missing data on individual variables included in the composite.

### Statistical analyses

Univariate comparisons of categorical variables were performed using Pearson’s chi-square (χ^2^) or Fisher’s exact test as applicable, and continuous variables using the Mann–Whitney U-test due to their non-normal distribution. Categorical variables are reported as frequencies *n* (%), and continuous data are reported as median with interquartile range (IQR). Each analysis was stratified by sex. The proportions of missing FH data among patients and controls, and among males and females, were compared to evaluate the impact of missing data on the analyses. Missing FH data were not imputed.

Univariable and multivariable logistic regression were applied to analyse the association between FH and eCIS. The primary analysis assessed the two pre-specified composite variables: FH among FDR and FH among grandparents. The multivariable model was adjusted for those stroke risk factors that showed a significant association (*P* < .05) with eCIS in the univariate comparisons. In the regression analyses, we excluded those independent variables with fewer than five observations per group to avoid unstable estimates. Participants with missing variable data were excluded listwise in each model. A Z-test was used to evaluate heterogeneity in effect sizes between males and females.

Statistical analyses were conducted using IBM SPSS Statistics version 29.0 (IBM Corp., Armonk, NY, USA). A two-sided *P* < .05 was considered statistically significant. Multiple testing correction was not applied because the variables cannot be regarded as independent, and the primary analysis used only 2 compound variables (ie, FH among FDR and FH among grandparents).

## Results

### Baseline characteristics

We included 508 eCIS patients and 520 controls after excluding 99 patients and 87 controls due to missing FH information. Table 1 compares demographic details and cardiovascular comorbidities between patients and controls. Patients more often had a history of CVD, hypertension, diabetes mellitus, current smoking, abdominal obesity, physical inactivity, heavy alcohol use, poor diet, stress, depression and low education compared to controls.

**Table 1:** Demographic characteristics and comorbidities of the study population

Characteristic	Patient, *n*/*N* (%)	Control, *n*/*N* (%)	*P*-value
**Age, median (IQR), year**	41 (34–46)	41 (33–46)	0.49
**Sex, female**	236/508 (46)	245/520 (47)	0.83
**Low education**	282/507 (56)	181/520 (35)	<0.001
**Previous CVD**	17/508 (3)	6/520 (1)	0.02
**Hypertension**	178/508 (35)	140/517 (27)	0.01
**Diabetes mellitus**	15/508 (3)	10/519 (2)	0.29
**Hypercholesterolemia**	13/508 (3)	25/518 (5)	0.05
**Current smoking**	162/505 (32)	78/519 (15)	<0.001
**Abdominal obesity**	299/508 (59)	229/515 (44)	<0.001
**Physical inactivity**	145/504 (29)	116/517 (22)	0.02
**Heavy alcohol use**	69/505 (14)	34/519 (7)	<0.001
**Poor diet**	265/505 (52)	189/520 (36)	<0.001
**Stress**	256/507 (50)	214/520 (41)	<0.001
**Depression**	151/507 (30)	120/515 (23)	0.02

All data are presented as *n* (%) unless otherwise indicated.

Abbreviations: CVD = cardiovascular disease; IQR = interquartile range.

### Univariate comparison of family history between patients and controls

Table 2 and Table S2 show the proportion of eCIS patients and controls reporting a positive FH of a cardiovascular disease. eCIS patients were more likely than controls to report a positive FH of stroke in FDR (20% vs 14%, *P =* .01) and in grandparents (47% vs 39%; *P =* .01). There were few differences between the proportions of patients and controls reporting family histories of VTE, CAD and aneurysms, although the numbers of events reported in family members were smaller.

**Table 2:** Univariable comparison of family history of cardiovascular diseases between patients and controls

Characteristic	Patient, *n*/*N* (%)	Control, *n*/*N* (%)	*P*-value
**FH of stroke**			
**FDR**	99/505 (20)	71/516 (14)	.01
**FDR, at age < 50 years**	25/505 (5)	11/516 (2)	.01
**Grandparents**	203/432 (47)	180/466 (39)	.01
**Grandparents, at age < 50 years**	12/417 (3)	5/461 (1)	.05
**FH of VTE**			
**FDR**	61/502 (12)	52/515 (10)	.30
**FDR, at age < 50 years**	24/501 (5)	8/515 (2)	.003
**Grandparents**	68/380 (18)	69/426 (16)	.52
**Grandparents, at age < 50 years**	4/371 (1)	2/420 (0)	.43
**FH of CAD**			
**FDR**	122/504 (24)	116/516 (22)	.51
**FDR, at age < 50 years**	17/501 (3)	12/516 (2)	.31
**Grandparents**	217/403 (54)	249/457 (54)	.85
**Grandparents, at age < 50 years**	22/375 (6)	18/436 (4)	.25
**FH of aneurysm**			
**FDR**	17/501 (3)	19/515 (4)	.80
**FDR, at age < 50 years**	6/500 (1)	5/515 (1)	.72
**Grandparents**	27/386 (7)	25/451 (6)	.39
**Grandparents, at age < 50 years**	4/385 (1)	4/448 (1)	>.99

Abbreviations: CAD = coronary artery disease; FDR = first-degree relative; FH = family history; VTE = venous thromboembolism.

The proportion of missing FH data ranged from 4% to 17% regarding FDR and from 22% to 55% regarding grandparents when comparing patients and controls (Table S3). There were no statistically significant differences in the proportions of missing data between patients and controls regarding FH of stroke among FDR or FH of VTE among FDR, except for VTE among mothers. Patients had more missing data than controls when reporting FH of CAD, except for offspring and siblings, and when reporting FH of aneurysms, except for offspring. Except for VTE among paternal grandmothers, patients were less often able to report FH among their grandparents compared to controls. Regarding sex differences in FH reporting, male participants more frequently could not report FH among their maternal grandparents, while female participants more often could not report FH of aneurysms among their fathers (Table S4). Otherwise, the proportions of missing data did not differ between men and women.

### Multivariable regression

**Figure 1:**
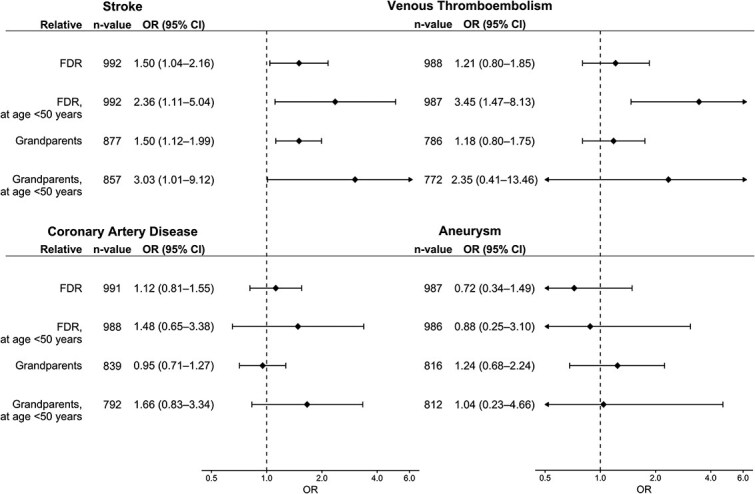
Association of a family history of stroke, venous thromboembolism, coronary artery disease and aneurysms with early-onset cryptogenic ischaemic stroke. Data are from multivariable adjusted logistic regression models. Abbreviations: OR = odds ratio, CI = confidence interval; FDR = first-degree relatives.

Results of the primary multivariable analyses are summarized in Figure 1. eCIS was significantly associated with a FH of stroke in FDR (OR 1.50; 95% CI, 1.04–2.16) and grandparents (1.50; 1.12–1.99). FH of VTE, CAD or aneurysms was not associated with eCIS in the multivariable model. After stratification by sex, variables did not show a significant heterogeneity between the sexes. Results regarding FH of each family member separately are presented in Table S5.

### Family history of early-onset cardiovascular disease

Table 2 and Table S6 present univariate comparisons of the association between FH of early-onset CVD and eCIS, and Figure 1 and Table S7 summarize the results of multivariable logistic regression. FH of early-onset stroke in FDR (2.36; 1.11–5.04) and grandparents (3.03; 1.01–9.12) showed an association with eCIS. FH for early-onset VTE in FDR (3.45; 1.47–8.13) was associated with eCIS. FH of early-onset CAD or aneurysms among FDR or grandparents did not show an association with eCIS.

### Stratification by high-risk patent foramen ovale

**Figure 2:**
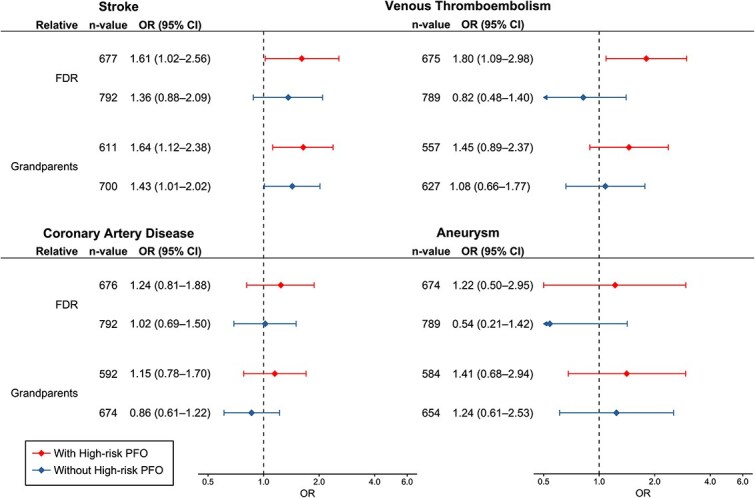
Association of a family history of stroke, venous thromboembolism, coronary artery disease and aneurysms with early-onset cryptogenic ischaemic stroke by patient’s high-risk patent foramen ovale status. Data are from multivariable adjusted logistic regression models. Abbreviations: OR = odds ratio, CI = confidence interval; FDR = first-degree relatives.

Figure 2 and Table S8 summarize results stratified by HR-PFO status. Information on HR-PFO was available for 98% (*n* = 499) of patients, of whom 36% (*n* = 182) had HR-PFO. Information on PFO was missing in 2% (*n* = 9) of patients, who were therefore excluded from the analysis. Information on PFO status of controls was not collected systematically at all study sites, so all controls with available FH data were used as the comparison group. When comparing HR-PFO patients to stroke-free controls, the association between eCIS and FH of stroke among FDR (1.61; 1.02–2.56) and grandparents (1.64; 1.12–2.38) was significant. When comparing patients without HR-PFO to controls, only FH of stroke in grandparents showed a significant association (1.43; 1.01–2.02). In HR-PFO patients, FH of VTE in FDR was associated with eCIS (1.80; 1.09–2.98). FH of CAD or aneurysms was not associated with eCIS in either HR-PFO patients or among patients without HR-PFO.

## Discussion

In this large multicentre case–control study including over 500 eCIS patients and matched controls, eCIS was independently associated with a FH of stroke. The association was stronger when considering the FH of early-onset stroke (<50 years). Furthermore, we identified a robust association between eCIS and FH of early-onset VTE.

Patients with eCIS have historically represented a minority in studies examining the association between stroke and FH of stroke. A Swedish study involving 162 cryptogenic ischaemic stroke patients under the age of 70 found an independent association between cryptogenic ischaemic stroke and FH of stroke.^7^ Similarly, a smaller Greek study reported a positive association with FH of stroke among a cohort of 57 cryptogenic ischaemic stroke patients of all ages.^8^ Our study, which includes a substantially larger number of eCIS patients under the age of 50, significantly strengthens the existing evidence supporting FH of stroke as an independent risk factor for eCIS.

In our cohort, FH for early-onset VTE was associated with eCIS. Previous observational studies have not consistently confirmed a link between familial susceptibility to VTE and stroke,^17^ despite genetic studies supporting an association between early-onset ischaemic stroke and VTE.^18^ Moreover, inherited thrombophilia disorders, such as factor V Leiden, prothrombin G20210A mutation, protein C deficiency and protein S deficiency, have been associated with increased risk of arterial ischaemic strokes,^19^ and are thought to play a role in eCIS.^20^ Our findings, in conjunction with prior evidence, underscore the potential contribution of familial prothrombotic factors to the risk of eCIS and warrant further investigation into these mechanisms.

In our study, FH of CAD was not associated with eCIS. Previous studies have reported a positive association with FH of CAD and ischaemic stroke in populations under 70 years old,^7^^,^^21^ and genetic studies also support this link.^22^ However, in agreement with our results, the Swedish study did not show an association between cryptogenic ischaemic stroke and CAD when considering specific stroke subtypes.^7^ Thus, ours and others’ findings suggest that familial risk of CAD might not substantially increase the risk of eCIS.

In the present study, FH of aortic or cerebrovascular aneurysms did not show an association with eCIS. Despite some reports suggesting that FH of stroke other than subarachnoid haemorrhage might increase the risk of the presence of an unruptured intracranial aneurysm, our study does not provide evidence for shared familial risk of aneurysms and eCIS.^23^ To our knowledge, no previous study has addressed the association between FH of aneurysms and eCIS.

We also performed sex-stratified analyses, where we found no heterogeneity. The Norwegian Stroke in the Young Study found young female stroke patients to be more likely to report a positive FH of cardiovascular disease.^24^ In contrast, our results do not support a significant role for sex differences in the association of FH of CVD and eCIS, however, our study may be underpowered to detect a possible difference. Larger studies should be conducted to make definitive conclusions.

The analyses stratified by patients’ HR-PFO revealed that the association between FH and stroke was stronger among HR-PFO patients than among non-HR-PFO patients, although we could not perform a statistical comparison of effect sizes between the two groups owing to shared controls in this analysis. More importantly, while FH of VTE (any age) did not show a significant association in the primary analysis, among HR-PFO patients, FH of VTE among FDR was associated with eCIS. The association between FH of VTE and eCIS only in the presence of HR-PFO, as shown in this study, highlights the need for further research in identifying occult prothrombotic risk in patients with eCIS.

We comprehensively analysed the proportion of missing data. The FH of grandparents had more missing data than the FH of FDR. Patients had more missing data than controls, especially regarding grandparents. Males had less knowledge of their maternal grandparents than females. Previously, knowledge about stroke patients’ FH has been studied in the Norwegian Stroke in the Young Study. In our cohort, 5% and 9% of patients had missing FH information for stroke in mothers and fathers, respectively, compared with 4% and 7% in the Norwegian cohort. Regarding FH of stroke among grandparents, 31%–43% of patients had missing data in our cohort compared to 32%–49% in the Norwegian study.^24^

### Strengths and limitations

Our study has several strengths. SECRETO is a comprehensive, multicentre study with clearly defined inclusion and exclusion criteria and careful phenotypic assessment, including patients’ HR-PFO.^14^ All data were collected prospectively. FH information was assessed using a structured questionnaire, improving the consistency and completeness of FH data. In addition to stroke, we also covered the FH of other CVDs. Nevertheless, our study has limitations that should be considered. Most importantly, our FH data relied solely on participants’ self-reports rather than verified medical records. This potentially introduces recall bias or inaccuracies due to limited familial communication or a lack of medical background knowledge. There is also a risk of differential recall between cases and controls. However, given that patients had more missing FH data than controls, we believe the effect of potential recall bias would likely dilute rather than inflate the effect estimates.

Regarding FH of stroke, we could not differentiate between FH of ischaemic strokes and haemorrhagic strokes or specific ischaemic stroke subtypes, which increases the heterogeneity of the exposure and likely could attenuate the effect estimates. However, our approach is in line with previous studies on FH of stroke, which have also used an aggregate across all stroke types.^7^^,^^8^ Also, our sample size remains too limited for consideration of the FH of specific stroke subtypes. Further studies with larger sample sizes are needed to explore the FH of specific stroke subtypes.

The proportion of missing data was noticeable, especially regarding the FH of grandparents. This was, however, anticipated, given that information on grandparents is often subject to limitations related to the likelihood of them being deceased and the incomplete transmission of medical information over time.

There is also an inherent risk of type 1 error owing to the relation of the individual FH variables tested. To mitigate this risk, compound variables incorporating FH among FDR and grandparents were used. We chose not to apply a multiple-comparison correction because the analyses were hypothesis-driven and the variables were correlated. Applying such a correction would likely be overly conservative and could obscure true associations.

Finally, we acknowledge that FH cannot be directly regarded as evidence of genetic risk, considering that non-genetic factors, such as social determinants and shared environment, also aggregate within families.^21^^,^^25^ Future studies should address how lifestyle risk factor clustering modifies the association between FH and eCIS.

## Conclusion

The association between FH of stroke and eCIS warrants further studies to assess the genetic contribution to eCIS. The associations between eCIS and FH of VTE—particularly among patients with HR-PFO and those with FH of early-onset VTE—support the potential role of heritable prothrombotic factors in eCIS, especially in the presence of HR-PFO. Our study provides initial backbone for assessing FH of stroke or VTE to inform decision-making in primary prevention, screening for thrombophilia and risk stratification of PFO-associated stroke.

## Supplementary Material

Supplementary_tables_final_280226

## Data Availability

Data will be provided upon reasonable request to the corresponding author.
